# New Anti‐Fibrotic Strategies for Keloids: Insights From Single‐Cell Multi‐Omics

**DOI:** 10.1111/cpr.13818

**Published:** 2025-02-04

**Authors:** Songyun Zhao, Jiaheng Xie, Qian Zhang, Tianyi Ni, Jinde Lin, Weicheng Gao, Liping Zhao, Min Yi, Liying Tu, Pengpeng Zhang, Dan Wu, Qikai Tang, Chenfeng Ma, Yucang He, Liqun Li, Guoping Wu, Wei Yan

**Affiliations:** ^1^ Department of Plastic Surgery The Affiliated Friendship Plastic Surgery Hospital of Nanjing Medical University Nanjing China; ^2^ Department of Plastic Surgery The First Affiliated Hospital of Wenzhou Medical University Wenzhou China; ^3^ Department of Plastic Surgery, Xiangya Hospital Central South University Changsha China; ^4^ Department of General Surgery The First Affiliated Hospital of Nanjing Medical University Nanjing China; ^5^ Department of Burn and Plastic Surgery The First Affiliated Hospital of Nanjing Medical University Nanjing China; ^6^ Department of Lung Cancer Surgery Tianjin Medical University Cancer Institute and Hospital Tianjin China; ^7^ Department of Dermatology Huashan Hospital of Fudan University Shanghai China; ^8^ Department of Neurosurgery The First Affiliated Hospital of Nanjing Medical University Nanjing China

**Keywords:** anti‐fibrotic therapy, fibroblasts, keloid, scRNA‐seq, spatial transcriptomics, TGF‐β

## Abstract

Keloids are complex pathological skin scars characterised by excessive growth of fibrous tissue and abnormal accumulation of extracellular matrix (ECM). Despite various treatment options available, the treatment of keloids remains a major clinical challenge due to high recurrence rates and inconsistent therapeutic outcomes. By collecting three keloid tissues and three normal skin samples and utilising single‐cell RNA sequencing (scRNA‐seq), we delved into the cellular heterogeneity and molecular mechanisms of keloids. Our study identified multiple fibroblast subpopulations within keloid tissue. Enrichment and cell–cell communication analyses revealed that POSTN‐positive mesenchymal fibroblasts (POSTN+ mesenchymal fibs) are more prevalent in keloids and exhibit higher transforming growth factor β (TGF‐β) signalling activity, potentially playing a central role in excessive fibrosis. In contrast, IGFBP2‐positive fibroblasts (IGFBP2+ fibs) are more abundant in normal skin, insensitive to TGF‐β and Periostin signalling, and possess anti‐fibrotic potential, possibly related to limited tissue repair and regenerative capacity. Trajectory analysis inferred the differentiation states and patterns of different fibroblast subpopulations. Additionally, we explored the heterogeneity of endothelial cells, finding an endothelial cell subpopulation (EC10) exhibiting mesenchymal activation characteristics, which may work with specific fibroblasts to promote abnormal angiogenesis and endothelial‐to‐mesenchymal transition processes. Spatial transcriptomics analysis has shown that the proportion of IGFBP2+ fibroblasts relatively increases in acne keloidalis after hormonal treatment, further demonstrating their value as potential therapeutic targets. Ultimately, we separated these two subpopulations using flow cytometry, highlighting their opposing roles in the secretion of the ECM. These findings provide new insights into the pathogenesis of keloids and lay the theoretical foundation for the development of innovative anti‐fibrotic treatment strategies.

## Graphical abstract

Our research not only revealed the ubiquity and activity of POSTN‐positive mesenchymal fibroblasts in keloids but also discovered the predominant role of IGFBP2‐positive fibroblasts in normal skin and their potential significance in the process of anti‐fibrotic therapy. Through these findings, we have provided a new perspective for the treatment of keloids and laid the theoretical foundation for the development of innovative anti‐fibrotic therapeutic strategies.

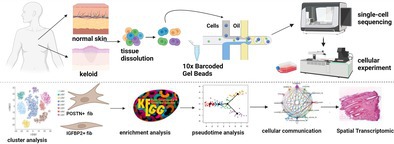

## 1 Introduction

Keloids are a unique type of pathological skin scar characterised by dysregulated fibrous proliferation, excessive accumulation of extracellular matrix (ECM), abundant angiogenesis, and lesions that extend beyond the original skin injury, continuing to grow persistently [1]. Epidemiological studies have shown significant differences in the incidence of keloids among various ethnic groups, with individuals of yellow or black skin being more susceptible. Moreover, those with a personal or family history of keloids have a higher propensity for the condition [2, 3]. Clinically, keloids typically form within months after an injury and can rise about 0.5 cm above the skin surface. Notably, any form of skin injury, even minor acne, can lead to keloid formation [4]. This condition not only causes physiological symptoms such as itching and pain but also results in a range of psychosocial issues due to its appearance, significantly affecting patients' quality of life [5].

Statistical data indicate that the incidence of keloids varies significantly among different races, with the highest occurrence in black populations, reaching 5%–10%, and yellow populations following with a rate of 0.1%–1.0%. This has led to the continuous expansion of the keloid treatment market, which is projected to exceed 15 billion USD globally by 2031. This market growth is primarily driven by the increasing demand for effective treatment solutions. Current treatment strategies for keloids include surgical excision, injection therapies, and laser treatments. However, these treatments often face high recurrence rates and inconsistent efficacy [6, 7]. For instance, corticosteroids are among the most commonly used methods for preventing and treating keloids but may cause side effects such as hypopigmentation/hyperpigmentation, atrophy, and telangiectasia [8]. Given the limitations of existing treatments, effective management of keloids remains a significant challenge in clinical medicine. Therefore, the significance of this study lies in its focus not only on the basic biological characteristics of keloids but also on the exploration of potential new therapeutic targets, providing new perspectives and possible solutions for improving this common and far‐reaching skin pathology.

Fibroblasts are highly specialised cells derived from mesenchymal stromal cells and are widely distributed across various human tissues, including the gastrointestinal tract, bone marrow, skin, adipose tissue, and the epithelial lining of the lungs [9]. The fibroblasts are indispensable for the synthesis and upkeep of connective tissue that is replete with ECM, a component that is paramount for the proper physiological functioning of a multitude of organs. These cells assume a notably significant role in pivotal physiological processes, including wound healing, tissue remodelling, and immune regulation. Nonetheless, when subjected to perturbations by external factors such as mechanical trauma, exposure to toxins, infections, or autoimmune responses, the equilibrium of the tissue repair process may be disrupted. This disruption can precipitate an overabundance of ECM components, notably type I collagen, ultimately culminating in the formation of pathological scar tissue [10]. In keloids, a typical pathological condition, ECM components exhibit a characteristic parallel arrangement with significantly increased collagen production and a severe imbalance in the ratio of type I to type III collagen [11]. Various growth factors, such as platelet‐derived growth factor (PDGF), fibroblast growth factor β (FGF‐β), and insulin‐like growth factor I (IGF‐I), play important roles in promoting inflammatory responses and excessive ECM deposition [12]. As the central cell type in the process of skin fibrosis, fibroblasts demonstrate exceptionally strong proliferative potential, migratory and invasive capabilities, and ECM deposition ability. These characteristics are mainly regulated by fibrosis‐related growth factors such as transforming growth factor β (TGF‐β), FGF, PDGF, vascular endothelial growth factor (VEGF), and periostin (POSTN) [13, 14]. A deep understanding of fibroblast functions and their regulatory mechanisms is crucial for developing innovative therapeutic strategies for fibrotic diseases.

The rapid advancement of single‐cell RNA sequencing (scRNA‐seq) technology provides a powerful tool for in‐depth investigation of the cellular heterogeneity and molecular mechanisms of keloids. Compared to traditional bulk RNA sequencing, scRNA‐seq allows for unbiased transcriptomic analysis of tens of thousands of cells at single‐cell resolution, revealing the heterogeneity of cell populations and complex regulatory networks within keloid tissue [15, 16]. Studies have identified significant heterogeneity among fibroblasts in keloid tissue, which can be divided into several functionally distinct subpopulations. Trajectory analysis further elucidates the transition of fibroblasts from normal to pathological states, involving changes in gene expression patterns related to ECM production, inflammatory responses, and cell proliferation [17, 18]. To address the lack of spatial information in scRNA‐seq, researchers have combined it with spatial transcriptomics (ST) to explore the impact of cell–cell interactions and microenvironmental factors on mesenchymal fibroblasts in keloids [19]. These findings provide new insights into the pathological mechanisms of keloids and offer important clues for developing targeted therapeutic strategies, potentially improving the clinical management of keloids.

In this study, we integrate scRNA‐seq and ST technologies to deeply explore the cellular heterogeneity and molecular mechanisms of keloids. We aim to comprehensively reveal the cell types and functional characteristics within keloid tissue, with a particular focus on fibroblast subpopulations and their roles in disease progression. By comparing keloid and normal skin tissues, we hope to identify key cell communication pathways and signalling mechanisms, especially those with potential anti‐fibrotic properties. This multidimensional research approach is expected to provide new perspectives on the pathogenesis of keloids and lay a theoretical foundation for developing innovative anti‐fibrotic therapeutic strategies.

## 2 Materials and Methods

### 2.1 Patient Sources and Sample Preparation

This study was approved by the Medical Ethics Committee of the First Affiliated Hospital of Nanjing Medical University and the Affiliated Friendship Plastic Surgery Hospital (Approval No: 2024‐SR‐383). Informed consent was obtained from each patient before participation, and all participants were of Han ethnicity. We collected six skin tissue samples from patients undergoing skin plastic surgery at these hospitals. Three samples were keloid tissues from patients clinically diagnosed with keloids, and the other three were normal skin samples excised during plastic surgery. Supplementary Table 1 shows the clinical characteristics of all included patients. Tissue biopsies were collected from the central region of the keloids rather than the periphery. Before core excision, no patients had received chemotherapy, radiotherapy, or intralesional steroid treatment. We followed the same sample processing methods as in previous studies [20]. In short, after the removal of adipose tissue, the excised tissue was cut into small pieces with a diameter of 5 mm and incubated with Dispase II at 37°C for 2 h. Then, the samples were minced and digested with Collagenase IV at 37°C for 2 h. The cell suspension was filtered through a 70‐μm cell strainer and centrifuged at 400*g* for 10 min. After removing the supernatant, the pellet was washed with PBS at 400*g* for 5 min.

### 2.2 Library Preparation and Raw Data Processing

Utilising the Chromium Single Cell Library, Gel Bead & Multiplex Kit from 10× Genomics, we barcoded single‐cell suspensions for individual identification. Within the Chromium Controller instrument, these cells were encapsulated into gel beads, which were then subjected to lysis and barcoded reverse transcription. The resulting DNA libraries were sequenced on the Illumina HiSeq X platform with 150‐bp paired‐end reads. Raw sequencing reads were processed using Cell Ranger version 3.0.2 to generate comprehensive gene expression profiles, which were subsequently aligned to the GRCh38 reference genome. Gene and Unique Molecular Identifier (UMI) counts were performed using FeatureCounts software, thereby providing a detailed quantification of the transcriptome.

### 2.3 Preprocessing of scRNA‐seq

Analysis for subcluster and cell type annotation was conducted using the “Seurat” R package [21]. Briefly, cells that did not meet specific criteria were filtered out, and the remaining single‐cell transcriptomic expression matrix was integrated using the “harmony” R package. Highly variable genes were then selected for principal component analysis (PCA), and the top 30 significant principal components were used for Uniform Manifold Approximation and Projection (UMAP) dimensionality reduction. The “FindAllMarker” function identified differentially expressed genes (DEGs) in each cell subpopulation, and cell types and subtypes were annotated based on the expression of established marker genes for each cell type.

### 2.4 Source of ST and Deconvolution Algorithm

Acne keloidalis (AK) and keloids are both pathological skin scars, although they differ in aetiology, pathophysiology, and clinical presentation, they may share some biological characteristics, such as the potential to form raised scar tissue on the skin. Due to the lack of ST sequencing data for keloids, we collected previous data on AK before and after corticosteroid treatment [22]. The ST data were analysed using Seurat in R. We first normalised the UMI count data to eliminate the effects of sequencing depth and cell size. The “ScaleData” function was used to scale the data, reducing technical variation between different samples. The most variable features determined by the “SCTransform” function were used with the “RunPCA” function to perform PCA on the normalised and scaled data, reducing the dimensionality of the data and capturing the main variations. The “FindNeighbors” function constructed a graph based on nearest neighbours, and the “FindClusters” function identified different cell populations based on this graph. The “SpatialFeaturePlot” function was used to visualise the top 30 principal components, subpopulations, and genes. To infer cell types from ST data, we used the robust cell type decomposition (RCTD) method. RCTD is a deconvolution statistical method used to decompose cell‐type mixtures from ST data. It leverages cell type profiles from scRNA‐seq data to label pixels in ST through a supervised learning approach, fitting a statistical model to determine multiple cell types within each pixel [23]. We obtained weights for each cell type in each spot and determined the spatial localization of cell types based on these proportions.

### 2.5 Trajectory Analysis and Cell Stemness Inference

Using Monocle 3 to analyse single‐cell data, we first performed dimensionality reduction and clustering on the Seurat object, then converted it into a Monocle object to reconstruct differentiation trajectories [24]. We selected starting points from normal skin cell populations to trace their pseudo‐time trajectory toward keloid development. In this study, we used the “CytoTRACE” R package to infer cell differentiation states from scRNA‐seq data [25]. This package predicts the differentiation potential of cells by analysing gene count features, independent of specific time scales or continuous developmental processes.

### 2.6 Pathway Enrichment Analysis and Transcription Factor Prediction

To explore the biological significance of DEGs, we used the “clusterProfiler” R package for Gene Ontology (GO) and KEGG pathway enrichment analysis [26]. Metabolic activity was assessed using the “scMetabolism” algorithm [27]. Single‐cell regulatory network inference and clustering (SCENIC) aids researchers in understanding cell function and life activities by analysing the expression and activity of transcription factors (TFs), revealing regulatory mechanisms underlying cellular heterogeneity [28]. SCENIC uses the GENIE3 or GRNBoost algorithms to infer co‐expression relationships between TFs and target genes based on gene expression data. Finally, we used a gene‐motif ranking from the RcisTarget database (hg19‐TSS‐centric‐10 kb) to detect transcription start sites and gene regulatory networks in scRNA‐seq data from keloids.

### 2.7 Cellular Communication

In this study, we utilised the “CellChat” R package to conduct an in‐depth analysis of scRNA‐seq and ST data to uncover cellular communication networks [29]. We first imported the CellChat database, which contains ligand–receptor interaction information supported by the literature. We preprocessed the expression data to identify overexpressed ligands or receptors in each cell group and projected the gene expression data onto the protein–protein interaction network. After data preparation and pathway switching, we analysed the cellular communication network of the samples. This included identifying overexpressed genes, recognising overexpressed ligand–receptor pairs, calculating communication probabilities, and aggregating cell–cell communication. By providing tools for inferring and visualising interactions, such as the “netVisual_aggregate” and “netVisual_bubble” functions, we gained insights into the interaction relationships between different cell types in keloids and hypertrophic scars, thereby revealing patterns and dynamics of cellular communication within the biological system.

### 2.8 Immunofluorescence Staining

Paraffin‐embedded sections of keloid and normal skin tissues were subjected to immunofluorescence staining to assess the expression of IGFBP2 and APOD. Tissue sections were first baked at 60°C for 1 h to remove the paraffin, followed by deparaffinisation with xylene three times, 10 min each. The sections were then rehydrated through a gradient of ethanol (100%, 95%, 75%) for 5 min each and washed with PBS buffer three times. The sections were permeabilized with 0.1% Triton X‐100 for 10 min and then blocked with 5% bovine serum albumin for 1 h. The sections were incubated overnight at 4°C with appropriately diluted primary antibodies for IGFBP2 (Thermo Fisher Scientific, PA5‐85534) (green) and APOD (Abcam, ab196569) (red). After primary antibody incubation, the sections were washed with PBS and incubated with fluorescent dye‐labelled secondary antibodies at room temperature for 1 h. Finally, the nuclei were counterstained with DAPI (Abcam, ab104139) (blue). Images were captured using a fluorescence microscope, with a scale bar of 200 μm for each staining channel (IGFBP2, APOD, DAPI) and the merged image.

### 2.9 Flow Cytometry Sorting

To sort different fibroblast subpopulations from keloid tissue, single‐cell suspensions were first stained with anti‐CD90 antibodies (Abcam, ab307736) to isolate CD90^+^ fibroblasts. Cells were incubated with the antibody for 30 min at room temperature, protected from light, and then sorted using flow cytometry to obtain the CD90^+^ population. Within the sorted CD90^+^ fibroblasts, CD266^+^/CD9^−^ mesenchymal fibroblasts were further isolated. Cells were stained with anti‐CD266 (Abcam, ab275375) and anti‐CD9 antibodies (Abcam, ab236630) for 30 min and then subjected to a second round of flow cytometry sorting to collect the CD266^+^/CD9^−^ subpopulation. The remaining cells after the second sort were stained with anti‐SLC7A2 (Thermo Fisher Scientific, #ANT‐103‐F‐50UL) and anti‐interleukin‐1 receptor (IL1R1) antibodies (R&D Systems, FAB269P). A third round of flow cytometry sorting was conducted to separate SLC7A2^+^/IL1R1^−^ fibroblasts. These remaining cells were considered other fibroblast subtypes.

### 2.10 Western Blot

To assess protein expression in sorted keloid fibroblast subpopulations, Western blotting was performed. Total protein from each group was extracted using RIPA lysis buffer containing protease and phosphatase inhibitors. Protein concentration was quantified using a BCA protein assay kit. Equal amounts of protein (20–30 μg) were loaded onto SDS‐PAGE gels and separated by electrophoresis, followed by transfer to PVDF membranes. Membranes were blocked at room temperature with TBS‐T containing 5% non‐fat milk for 1 h and then incubated overnight at 4°C with primary antibodies against target proteins such as IGFBP2, APOD, or other markers of interest. After washing with TBS‐T, membranes were incubated with HRP‐conjugated secondary antibodies at room temperature for 1 h. Protein bands were visualised using an enhanced chemiluminescence detection system and quantified using ImageJ software. GAPDH was used as a loading control for normalisation. The results were used to compare protein expression levels among different fibroblast subpopulations derived from keloid tissue. The antibodies used were as follows: IGFBP2 (Abcam, ab188200); APOD (Abcam, ab256496); POSTN (Abcam, ab315104); COMP (Abcam, ab300555); Collagen I (Abcam, ab138492); Collagen III (Abcam, ab184993); and GAPDH (Abcam, ab8245).

### 2.11 Real‐Time Quantitative PCR

Total RNA was extracted from samples using the TRIzol reagent (Invitrogen) following the manufacturer's guidelines for RNA isolation. Subsequently, first‐strand complementary DNA was synthesised using the PrimeScript RT reagent kit (Takara, Japan) by the provided protocol for reverse transcription. Gene‐specific primer pairs for amplification were designed using Primer Premier 5.0 software, ensuring primer specificity and efficiency. Quantitative real‐time PCR (qPCR) was conducted with SYBR Green Master Mix (Takara, Japan), strictly adhering to the manufacturer's instructions for qPCR. The qPCR reactions were set up and run under conditions specified by the protocol, including cycling parameters and data analysis settings. All primer sequences used in this study are listed in Supplementary Table 2.

### 2.12 Statistical Analysis

Statistical analysis was performed utilising R software version 4.2.1, encompassing both Student's *t* test and Wilcoxon test, with outcomes presented as the mean ± standard deviation. Adjustments to *p* values were made employing the false discovery rate approach. The visualisation of heatmaps and volcano plots within this study was enhanced with the aid of the “ClusterGVis” and “scRNAtoolVis” R packages, which can be accessed at their respective GitHub repositories (https://github.com/junjunlab/ClusterGVis, https://github.com/junjunlab/scRNAtoolVis). For a comprehensive solution in single‐cell data analysis, including bioinformatics visualisation, the “SCP” R package was instrumental, and it is available on GitHub (https://github.com/zhanghao‐njmu/SCP). All *p*‐values were determined through two‐tailed tests, establishing statistical significance at the threshold of *p* < 0.05.

## 3 Results

### 3.1 Single‐Cell Landscape of Keloid Patients

**FIGURE 1:**
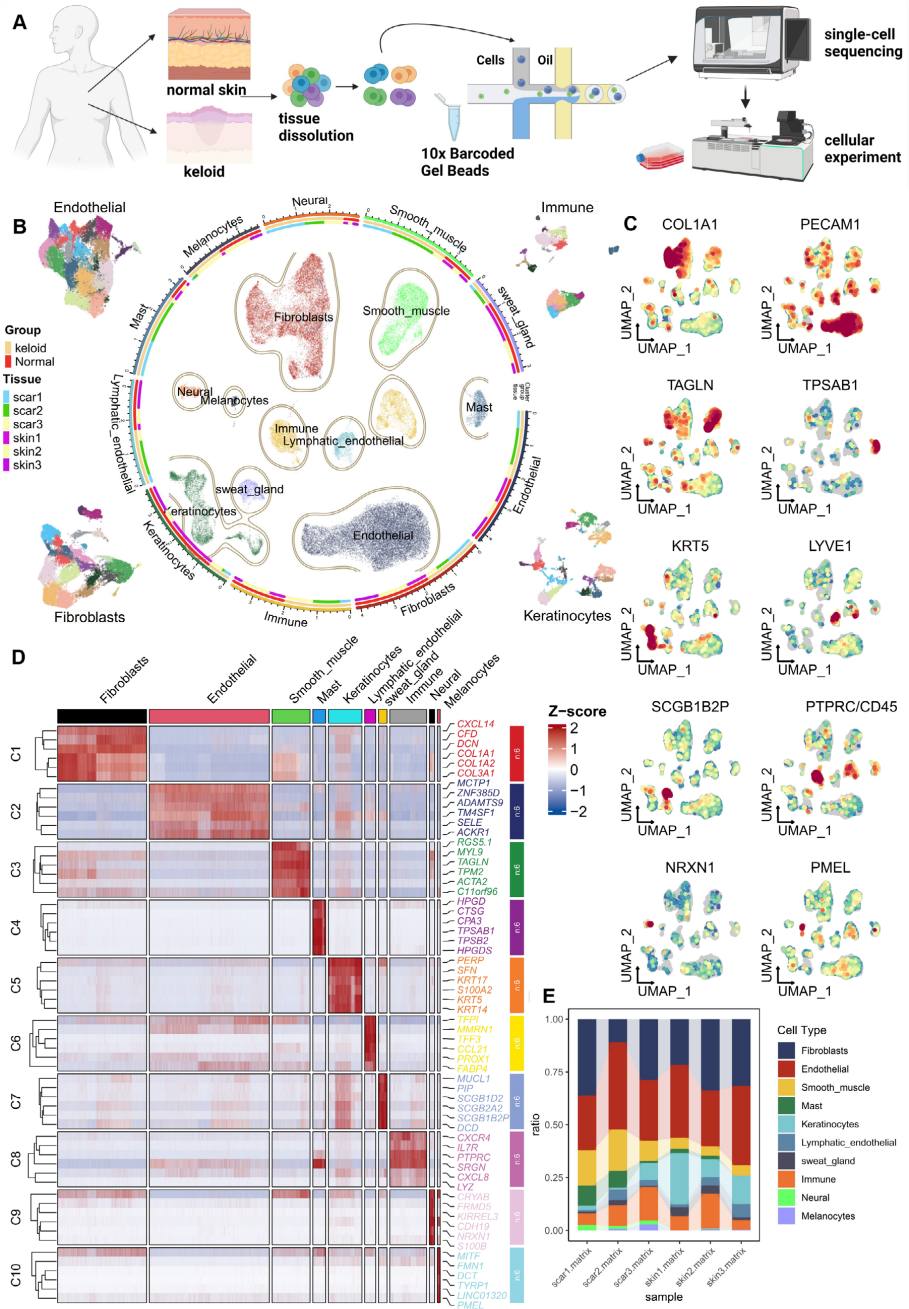
Single‐cell atlas of keloid nodule and normal skin cell composition. (A) Overview of the study design and workflow. Single‐cell suspensions from three keloid nodules and three normal control skin samples were collected, followed by scRNA‐seq on the 10× Genomics platform and subsequent cytological validation experiments. (B) UMAP plot of single‐cell features coloured by the 10 major cell types identified in this study. Distinct colours are used to differentiate between various groups and sample origins. (C) UMAP feature plots for typical marker genes of fibroblasts (COL1A1), endothelial cells (PECAM1), smooth muscle cells (TAGLN), mast cells (TPSAB1), keratinocytes (KRT5), lymphatic endothelial cells (LYVE1), sweat gland cells (SCGB1B2P), immune cells (PTPRC/CD45), neural cells (NRXN1), and melanocytes (PMEL). (D) Heatmap displaying the expression levels of major marker genes across all major cell types. Red indicates upregulated gene expression in that cell type, with the top 6 upregulated genes for each cell type highlighted. (E) Bar graph illustrating the relative proportions of each major cell subtype within each sample.

The design and workflow of all studies are briefly illustrated in Figure 1A. To elucidate cellular heterogeneity in the skin and explore the pathogenesis of fibrosis, we performed scRNA‐seq on dermal tissues from normal controls and keloid patients. After stringent quality control measures and unsupervised clustering, we obtained transcriptomes of 52,683 cells (keloid: 26,449; normal control: 26,234). Unsupervised UMAP clustering revealed 24 cell clusters with comparable expression patterns (Supplementary Figure 1A–C). Referring to several previous studies [20, 30], we validated each cluster as distinct cell populations using classical markers: fibroblasts (expressing COL1A1, COL1A2, and COL3A1); endothelial cells (expressing SELE, TM4SF1, and PECAM1); smooth muscle cells (expressing TAGLN, ACTA2, TPM2); mast cells (expressing TPSAB1); keratinocytes (expressing KRT5, KRT14, KRT10); lymphatic endothelial cells (expressing CCL21 and LYVE1); sweat gland cells (expressing SCGB1B2P and SCGB1D2); immune cells (expressing HLA‐DRA and PTPRC); neural cells (expressing NRXN1); and melanocytes (expressing TYRP1 and PMEL) (Figure 1B, Supplementary Figure 1E). Dot plots of cluster‐specific marker expression levels and proportions are shown (Supplementary Figure 1D), while UMAP plots illustrate the expression of specific marker genes in different cell types (Figure 1C). A heatmap displays the major marker genes for the 10 primary cell subtypes (Figure 1D). The cell lineage of normal samples versus keloids shows different relative cell number ratios (Figure 1E). An increased proportion of smooth muscle cells was primarily observed in keloid tissue, potentially related to collagen and ECM synthesis as well as inflammatory responses and tissue repair [1]. We further investigated the number of DEGs between keloid and normal samples. Results showed that neural cells and fibroblasts exhibited the highest number of upregulated genes, while smooth muscle cells and keratinocytes showed the highest number of downregulated genes. These cells demonstrated significant levels of transcriptional variability during scar formation (Supplementary Figure 1F).

### 3.2 Annotation and Functional Characterisation of Fibroblast Subpopulations

**FIGURE 2:**
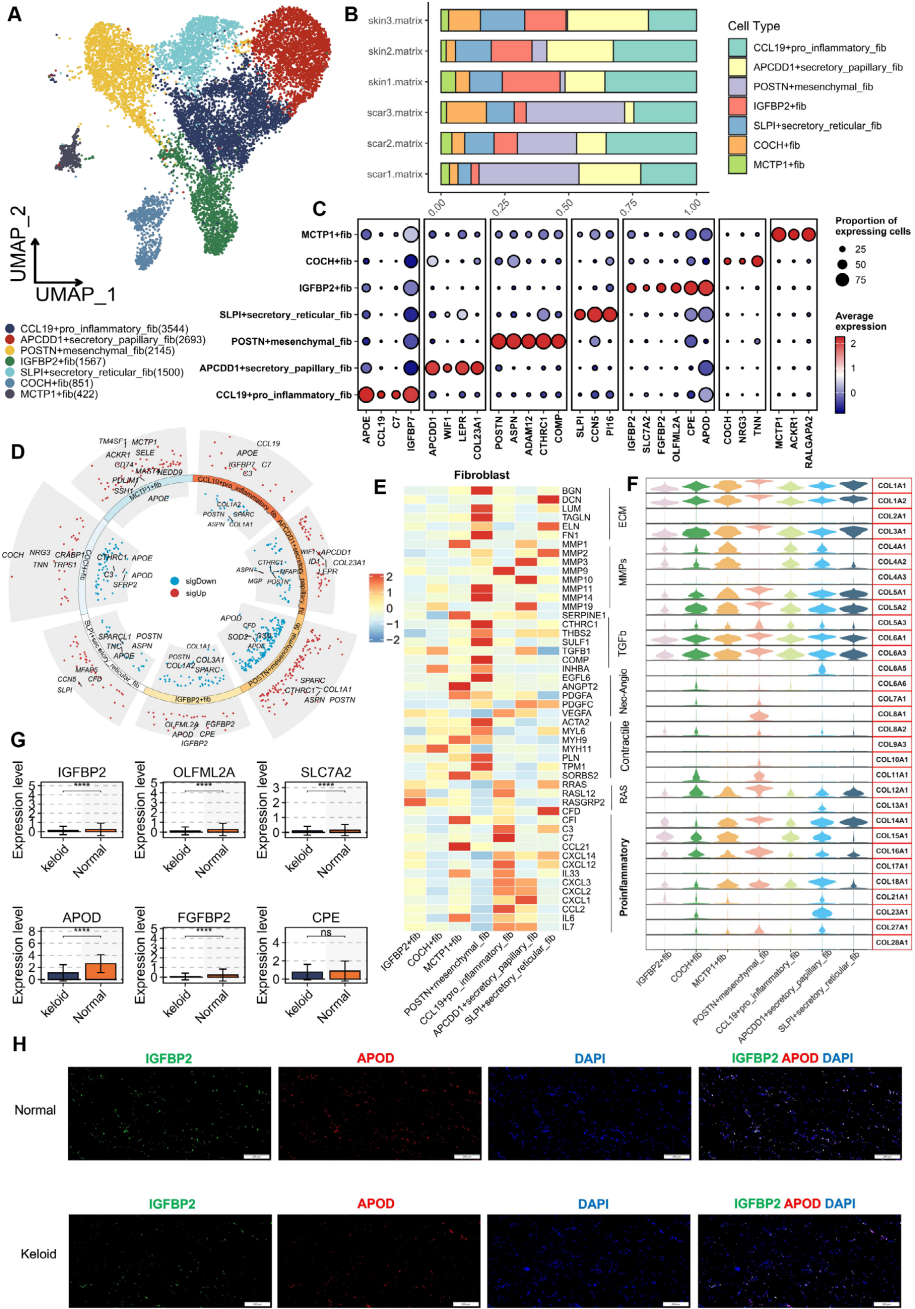
Heterogeneity of fibroblasts. (A) UMAP plot of seven distinct fibroblast subpopulations obtained through advanced clustering: CCL19+ pro‐inflammatory fibroblasts, APCDD1+ secretory papillary fibroblasts, POSTN+ mesenchymal fibroblasts, SLPI+ secretory reticular fibroblasts, IGFBP2+ fibroblasts, COCH+ fibroblasts, and MCTP1+ fibroblasts. (B) Bar chart showing the proportion of each of the seven fibroblast subtypes in the samples. (C) Dot plot highlighting the expression levels of major marker genes in the seven fibroblast subpopulations, including CCL19+ pro‐inflammatory fibroblasts (APOE, CCL19, C7, IGFBP7); APCDD1+ secretory papillary fibroblasts (APCDD1, WIF1, LEPR, COL23A1); POSTN+ mesenchymal fibroblasts (POSTN, ASPN, ADAM12, CTHRC1); SLPI+ secretory reticular fibroblasts (SLPI, CCN5, PI16); IGFBP2+ fibroblasts (IGFBP2, FGFBP2, OLFML2A, CPE, APOD, SLC7A2); COCH+ fibroblasts (COCH, NRG3, TNN); and MCTP1+ fibroblasts (MCTP1, ACKR1, RALGAPA2). (D) Circos plot illustrating all upregulated and downregulated genes in the seven fibroblast subpopulations. (E) Heatmap displaying the differential average expression levels of genes associated with common signalling pathways in the seven fibroblast subpopulations, including collagen, ECM, MMPs, TGF‐β, angiogenesis, contractility, RAS, and pro‐inflammatory. (F) Violin plot showing the expression levels of all collagen‐related genes in the seven fibroblast subpopulations. (G) In single‐cell RNA sequencing, the expression of six marker genes of IGFBP2+ fibroblasts (IGFBP2, FGFBP2, OLFML2A, CPE, APOD, SLC7A2) was compared between keloid and normal controls. (H) Normal and keloid tissues were immunofluorescently stained for IGFBP2 and APOD. The percentage of IGFBP2+/APOD+ cells in normal and keloid tissues was presented, with an observed decrease in the proportion of IGFBP2+/APOD+ in keloid tissues. Data are presented as mean ± standard deviation.

Fibroblasts are responsible for most collagen and ECM deposition in normal and abnormal wound healing, primarily driven by growth factors such as TGF‐β, PDGF, FGF‐β, and IGF‐I. In keloids, these growth factors enhance the scar phenotype of fibroblasts, becoming a core cause of keloid formation [1]. We reclustered fibroblasts and identified seven major clusters across six samples using UMAP (Supplementary Figure 2A–C). According to a previous study, dermal fibroblasts in normal individuals can be divided into four subgroups: secretory‐papillary, secretory‐reticular, mesenchymal, and pro‐inflammatory [17], which align with four of the seven subgroups we defined. Based on prior literature and marker genes for the seven clusters, we redefined the fibroblast subpopulations identified in the study: CCL19+ pro‐inflammatory fib (APOE, CCL19, C7, IGFBP7); APCDD1+ secretory papillary fib (APCDD1, WIF1, LEPR, COL23A1); POSTN+ mesenchymal fib (POSTN, ASPN, ADAM12, CTHRC1); SLPI+ secretory reticular fib (SLPI, CCN5, PI16); IGFBP2+ fib (IGFBP2, FGFBP2, OLFML2A, CPE, APOD, SLC7A2); COCH+ fib (COCH, NRG3, TNN); and MCTP1+ fib (MCTP1, ACKR1, RALGAPA2) (Figure 2A). These subtypes were defined based on the expression levels and proportions of marker genes (Figure 2C). Consistent with previous research, we compared differences between keloid mesenchymal fibroblasts and normal skin mesenchymal fibroblasts. Genes related to skeletal system development, ossification, and osteoblast differentiation were significantly increased in keloid samples (Supplementary Figure 2D). Notably, POSTN+ mesenchymal fib showed a higher expression proportion in keloid samples compared to normal ones. This aligns with previous findings suggesting that the microenvironment in keloid formation may contain specific signalling molecules and cell interactions promoting mesenchymal fibroblast proliferation and activation [20, 22]. Interestingly, we also found a lower proportion of IGFBP2+ fib in keloids, indicating a potentially overlooked subpopulation (Figure 2B). Supplementary Table 3 records the major DEGs for each cell type. Figure 2D presents the high and low expression DEGs across all fibroblast subpopulations. We screened classic fibroblast signalling pathway genes [31] and observed significant differences in pathway gene expression between IGFBP2+ fib and POSTN+ mesenchymal fib. POSTN+ mesenchymal fib notably expressed stronger TGF‐β and ECM‐related signals (Figure 2E). Specific collagen expression is related to specific fibroblast functions. We analysed collagen expression levels across the seven fibroblast subpopulations. Results showed that nearly all collagen genes were expressed at lower levels in IGFBP2+ fib (Figure 2F). We then focused on comparing the expression differences of IGFBP2+ fib marker genes (IGFBP2, FGFBP2, OLFML2A, CPE, APOD, SLC7A2) between keloids and normal controls. Except for CPE, other genes were more highly expressed in the normal group, especially APOD (Figure 2G). Overall, the low expression of IGFBP2+ fib in keloids suggests its role in pathological scar formation differs from other diseases. It likely plays a role in inhibiting excessive fibroblast proliferation and could be a promising target for anti‐fibrotic and anti‐proliferative therapies [32]. To validate scRNA‐seq findings, we performed immunofluorescence staining on normal controls and keloids. IGFBP2+ fib was identified by the expression of its two marker genes IGFBP2 and APOD (Figure 2H). Results showed a higher proportion of IGFBP2+/APOD+ cells in normal skin samples compared to the keloid group.

### 3.3 Signalling Enrichment and Biological Characteristics of Fibroblast Subpopulations

**FIGURE 3:**
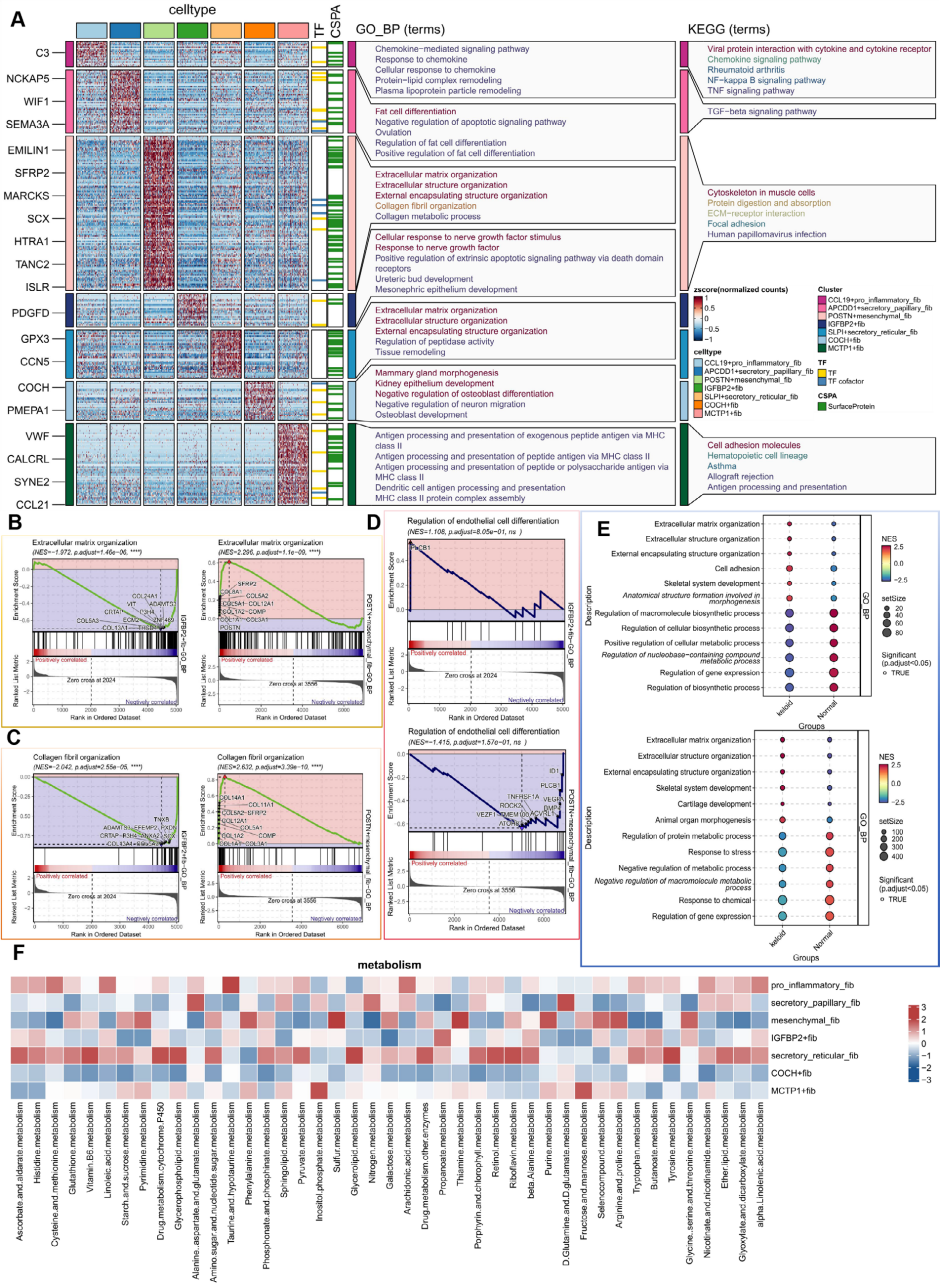
Enrichment analysis of fibroblasts. (A) The results of GO‐BP/KEGG enrichment analysis reveal differential genes, along with their corresponding transcription factors and surface proteins, across the seven fibroblast subpopulations. (B–D) Comparative GSEA is conducted between IGFBP2+ fibroblasts and POSTN+ mesenchymal fibroblasts with other fibroblasts, emphasising collagen fibre organisation, extracellular matrix organisation signalling, and endothelial cell differentiation regulatory signals. (E) The top five GSEA (GO‐BP) results are highlighted for IGFBP2+ fibroblasts and POSTN+ mesenchymal fibroblasts in both keloid and normal tissues. (F) A dot plot visually represents the activity of metabolic pathways within the seven fibroblast subpopulations.

We further explored the biological characteristics and enriched signalling pathways of fibroblasts with distinct gene transcription features. Enrichment analysis of marker genes from the seven fibroblast subpopulations revealed that POSTN+ mesenchymal fibs are primarily associated with biological processes related to the ECM and collagen fibril organisation in GO_BP, while IGFBP2+ fibs are mainly linked to responses to nerve growth factor and positive regulation of extrinsic apoptotic signalling pathways. In KEGG enrichment analysis, POSTN+ mesenchymal fibs were predominantly enriched in pathways related to cytoskeleton and ECM‐receptor interactions in muscle cells (Figure 3A). In GSEA, POSTN+ mesenchymal fibs showed a positive correlation with collagen fibril organisation and ECM organisation signals, whereas IGFBP2+ fibs showed the opposite trend (Figure 3B,C). Regarding intercellular signalling regulation, IGFBP2+ fibs actively regulated endothelial cell differentiation, while POSTN+ mesenchymal fibs exhibited opposite characteristics (Figure 3D). We then compared the signalling differences of these two cell types in keloid and normal tissues. Both POSTN+ mesenchymal fibs and IGFBP2+ fibs were associated with the ECM in keloids. In normal skin, IGFBP2+ fibs were linked to the positive regulation of biomolecule synthesis and cellular metabolism, whereas POSTN+ mesenchymal fibs were associated with the negative regulation of biomolecules (Figure 3E). Finally, given the different expression patterns of these fibroblast subpopulations in metabolism, we compared the activity scores across all metabolic pathways for these two subpopulations and assessed each cell's metabolic state using the AUCell method. Interestingly, POSTN+ mesenchymal fibs exhibited high purine metabolism activity, while IGFBP2+ fibs showed the opposite. Notably, IGFBP2+ fibs were more related to propionate metabolism pathways (Figure 3F).

### 3.4 Differentiation and Transcriptional Signalling of Fibroblast Subpopulations

**FIGURE 4:**
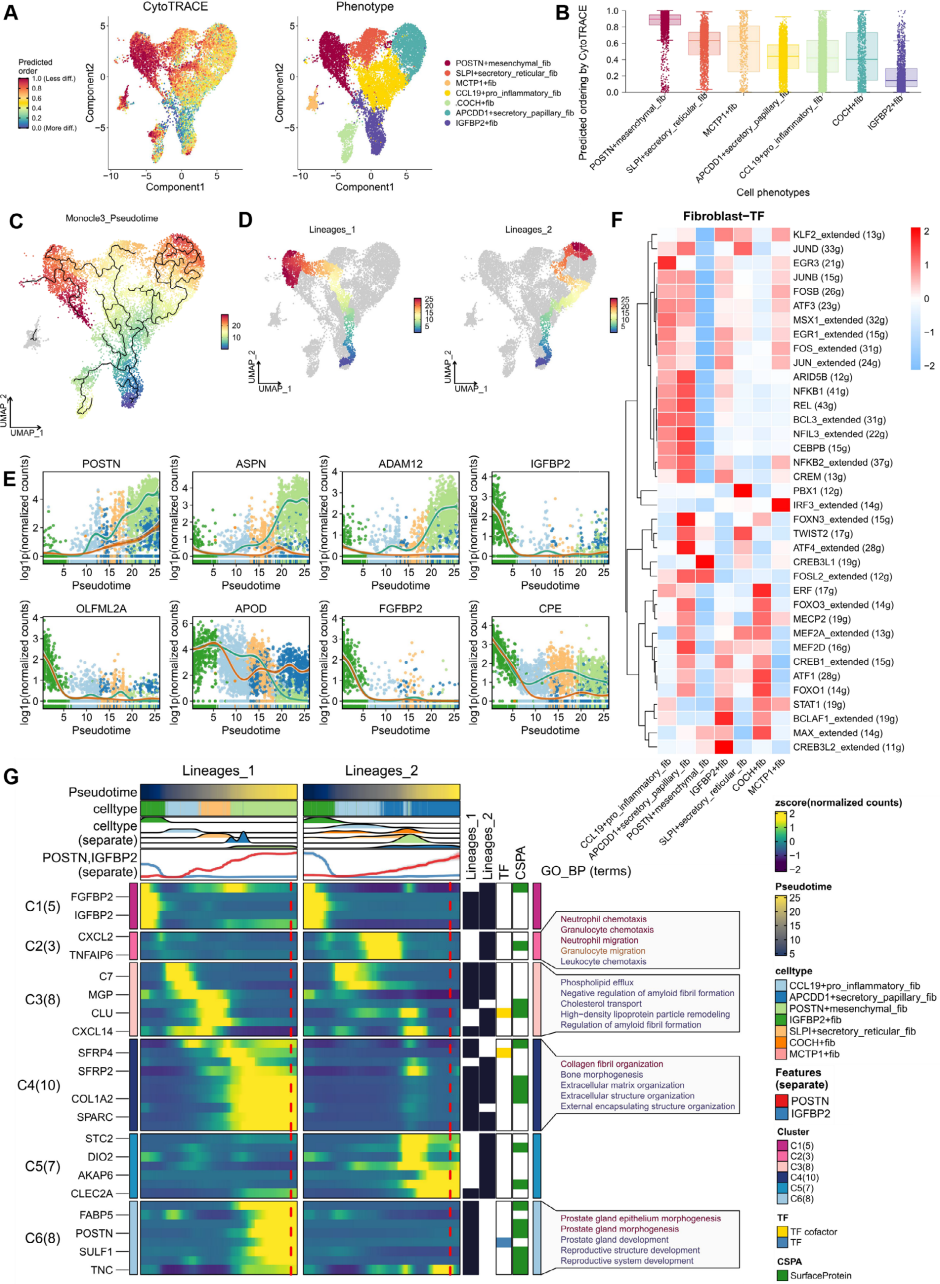
Differentiation trajectories of fibroblast subpopulations. (A, B) Stemness within the seven fibroblast subpopulations was assessed using CytoTRACE analysis, with higher CytoTRACE scores indicating increased stemness; statistical significance was evaluated using the Kruskal–Wallis test. CytoTRACE scores were mapped onto individual cells in a UMAP to provide a more intuitive representation of stemness variation across different fibroblast subpopulations. (C, D) Two pseudo‐time trajectories from the analysis reveal the differentiation paths of fibroblasts from their initial to terminal states. (E) Expression changes of marker genes for IGFBP2+ fibs and POSTN+ mesenchymal fibs along the two differentiation trajectories. POSTN+ mesenchymal fibs (POSTN, ASPN, ADAM12) and IGFBP2+ fibs (IGFBP2, FGFBP2, OLFML2A, CPE, APOD). (F) A heatmap illustrating the expression and activity of transcription factors (TFs) in the 7‐fibroblast subpopulations, as analysed by SCENIC. (G) Dynamic changes in cell types, GO‐BP enrichment results, corresponding transcription factors, and surface proteins along the two differentiation trajectories derived from Monocle 3 pseudo‐time analysis.

We evaluated the differentiation potential of various fibroblast subpopulations using CytoTRACE. Interestingly, POSTN+ mesenchymal fibs exhibited the highest stemness characteristics, while IGFBP2+ fibs had the lowest (Figure 4A,B). The high stemness of the POSTN+ mesenchymal fib subpopulation suggests these cells have greater self‐renewal and multipotent differentiation potential, likely related to keloid progression. In contrast, the low differentiation potential of IGFBP2+ fibs may be associated with limited tissue repair and regeneration capacity. To further clarify the differentiation of fibroblast states in keloids versus normal skin, we referenced methods from a recent study [19]. In Monocle 3, we selected IGFBP2+ fibs, which are more prevalent in normal cells, as the starting state. The pseudo‐time trajectory axis revealed terminal states at POSTN+ mesenchymal fib and APCDD1+ secretory papillary fib, indicating two distinct differentiation pathways from normal skin to keloids (Figure 4C,D). In the first differentiation pathway, marker genes of POSTN+ mesenchymal fib (POSTN, ASPN, ADAM12) steadily increased in expression, while the five marker genes of IGFBP2+ fib (IGFBP2, FGFBP2, OLFML2A, CPE, APOD) rapidly decreased (Figure 4E). The transition from IGFBP2+ fib to POSTN+ mesenchymal fib involves collagen fibril organisation and ECM generation (Figure 4G). Further TF predictions indicated that POSTN+ mesenchymal fib is primarily associated with CREB3L1 expression and regulatory activity (Figure 4F). In thyroid cancer, CREB3L1 mediates IL‐1α production to activate α‐SMA‐positive fibroblasts and downstream ECM signalling [33]. IGFBP2+ fib is mainly linked to BCLAF1 activity, which plays multifaceted roles in fibroblasts involving gene expression regulation, cell cycle, and apoptosis control, and responses to DNA damage [34].

### 3.5 Signalling Communication of Fibroblast Subpopulations

**FIGURE 5:**
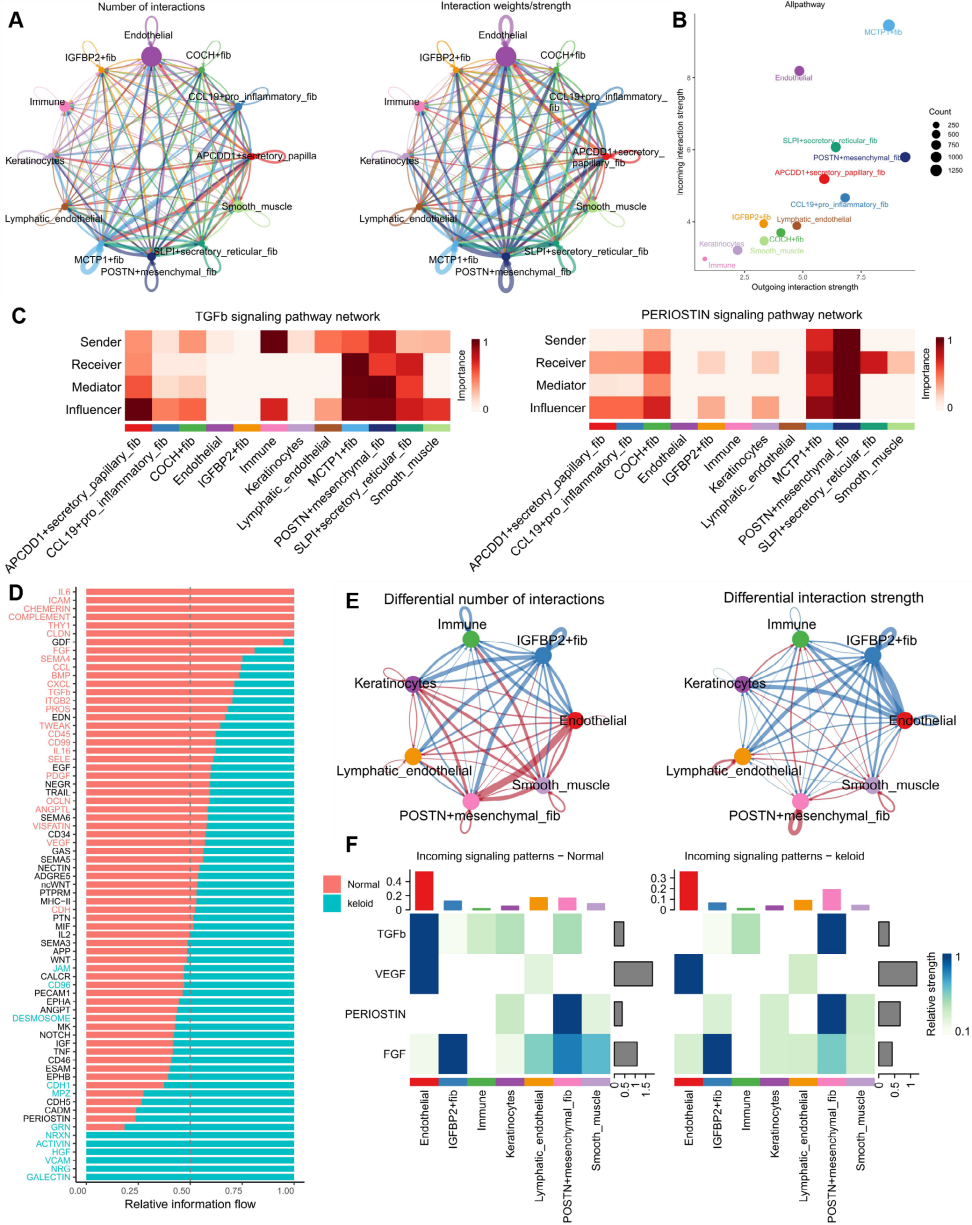
Cellular communication network. (A) Circular visualisation of the interaction strength and number between the seven fibroblast subpopulations and other cell types. (B) Input and output interaction strength across different cells. (C) Heatmap showing the main senders, receivers, mediators, and influencers in the TGF‐β and POSTN/Periostin signalling pathways inferred by network centrality scores. (D) Bar chart plotted in stacked mode ranking important signalling pathways based on differences in overall information flow between inferred keloid and normal skin networks; red pathways enriched in normal skin while green pathways enriched in keloids. (E) Differences in the number or intensity of interactions within cell–cell communication networks where red represents upregulated signals in keloids while blue represents those upregulated in normal skin. (F) Comparison of input signals across all cell types between keloid and normal skin networks.

Intercellular communication is a fundamental mechanism for information exchange and coordination among cells in multicellular organisms, often driving changes in cell states and functions. Figure 5A illustrates the intercellular communication strength and number between the seven fibroblast subtypes and other major cell types. Endothelial cells, MCTP1+ fibs, and POSTN+ mesenchymal fibs exhibited the highest input and output signalling strength, while immune cells, keratinocytes, and IGFBP2+ fibs showed lower intercellular signalling pathways (Figure 5B). TGF‐β plays a crucial role in tissue repair and regeneration, but persistent TGF‐β activity may lead to excessive fibrosis, resulting in scarring of the skin and visceral organs. Similarly, POSTN signalling is enhanced in mesenchymal fibroblasts, highly expressed in keloid and hypertrophic scar tissue, and positively correlated with TGF‐β1 expression [35]. Single‐cell analysis of TGF‐β signalling indicates that immune cells are the primary signal emitters in keloids, while MCTP1+ fibs and POSTN+ mesenchymal fibs mainly act as mediators and receivers. For POSTN/Periostin signalling, POSTN+ mesenchymal fibs are absolute participants. Interestingly, IGFBP2+ fibs exhibit the opposite behaviour to POSTN+ mesenchymal fibs, showing insensitivity to these signals (Figure 5C). Comparing signal flow between keloid and normal samples, TGF‐β signalling was even stronger in normal samples (Figure 5D). We then compared the signalling communication differences between the two fibroblast types in scar and normal samples. Figure 5E clearly shows that POSTN+ mesenchymal fibs dominate communication strength and number in keloid samples, whereas IGFBP2+ fibs play a leading role in normal samples. Finally, we compared the input signal strength of these cell subtypes in keloid and normal samples. Results indicated that POSTN+ mesenchymal fibs received more TGF‐β signals in scar tissue, while IGFBP2+ fibs received the least POSTN/Periostin signals (Figure 5F).

### 3.6 Mesenchymal Activation of Endothelial Cells and Fibroblast Signalling Communication

**FIGURE 6:**
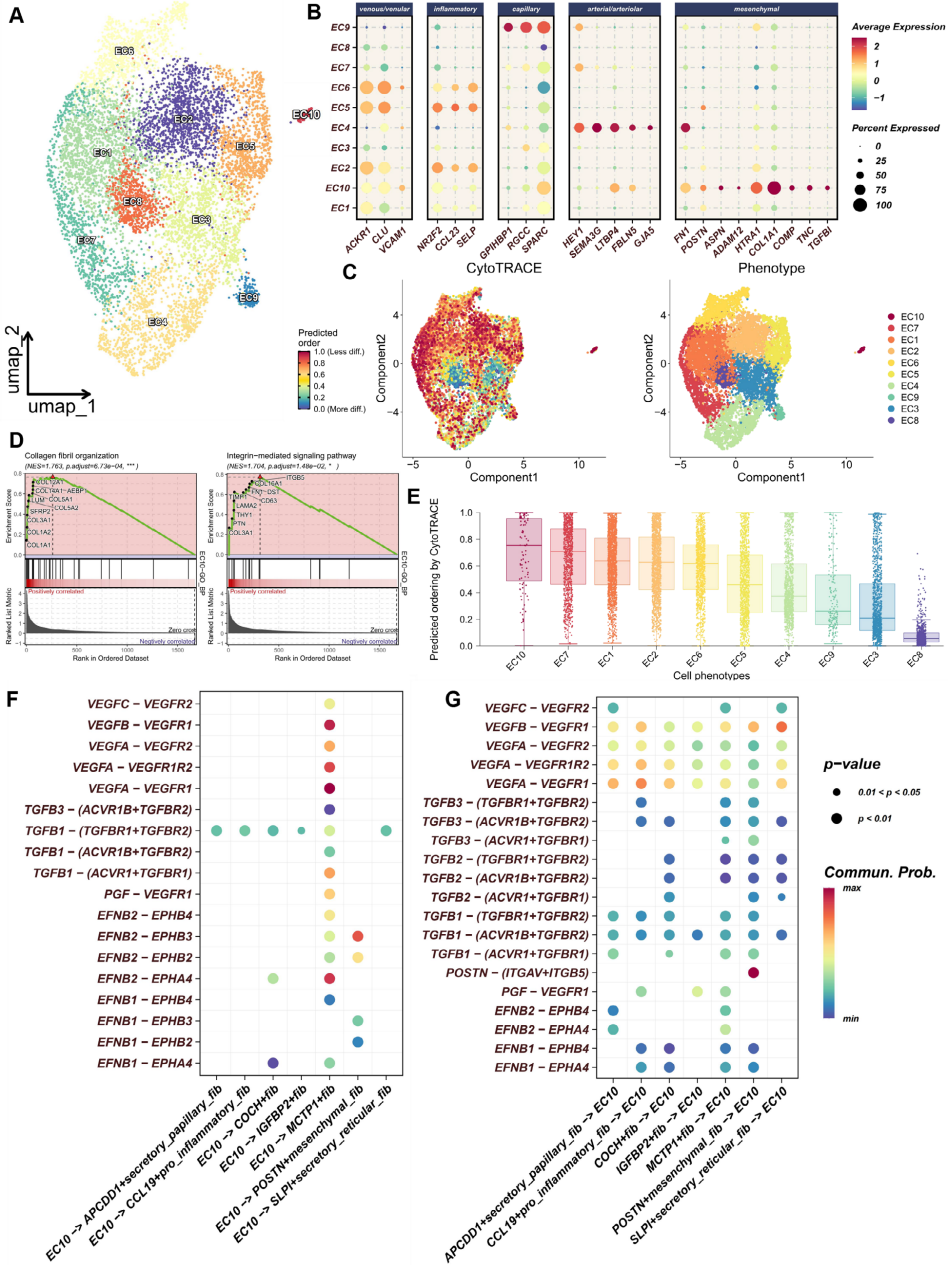
Endothelial cell heterogeneity. (A) UMAP plot of 10 endothelial cell clusters obtained from further clustering. (B) Dot plot showing the expression of vascular marker genes across the 10 endothelial cell subpopulations. (C, E) CytoTRACE analysis assessing stemness among the 10 endothelial cell subpopulations; higher CytoTRACE scores indicate increased stemness; CytoTRACE scores are mapped onto UMAP to visually represent changes among different endothelial cell subpopulations. (D) GSEA comparing EC10 (the 10th endothelial cell subpopulation) with other endothelial cells regarding collagen fibril organisation and integrin‐mediated signalling pathways. (F, G) Ligand–receptor information bubble plots between EC10 and the seven fibroblast subpopulations. Here, the focus is on intercellular signals that have been reported in keloids.

Shim et al. identified mesenchymal activation within endothelial cells in keloids through single‐cell sequencing and immunofluorescence experiments, with the relevant endothelial cell subpopulation characterised by the expression of POSTN, FN1, and HtrA serine peptidase 1 [19]. In Figure 3D, we observed through GSEA that IGFBP2+ fibs and POSTN+ mesenchymal fibs exhibit opposite regulatory effects on endothelial cell differentiation. Additionally, we found that both IGFBP2+ fibs and POSTN+ mesenchymal fibs display strong cellular communication signals with endothelial cells in both keloid and normal skin tissues (Figure 5E,F). We aimed to further explore potential mesenchymal activation signals and abnormal angiogenesis patterns between endothelial and fibroblast subpopulations. By reclustering, we identified 10 major endothelial cell clusters using UMAP (Figure 6A). A recent study categorised vascular endothelial cells in the skin into venous/vascular, capillary, arterial/arteriole, and inflammatory states. We collected all relevant markers to annotate the 10 endothelial cell subpopulations [36, 37]. We found that EC9 exhibited a capillary state, EC4 was more likely arterial/arteriole, and EC10 displayed transcriptional features of mesenchymal activation in keloids (Figure 6B). CytoTRACE indicated that both EC10 and POSTN+ mesenchymal fibs exhibited the highest stemness and differentiation potential (Figure 6C,E). GSEA results showed a positive correlation between EC10 and collagen fibril organisation as well as integrin‐mediated signalling pathways (Figure 6D). Finally, we used CellChat to investigate cellular communication and potential ligand–receptor interactions between these mesenchymal‐like endothelial cells and fibroblast subpopulations (Supplementary Figure 3A,B). We analysed all previously reported potential intercellular signals [30]. Interestingly, there was more cellular signalling between MCTP1+ fibs and EC10, including VEGF, TGF‐β, and EPHB pathways. This might explain the source of abnormal angiogenesis patterns in keloids. In contrast, IGFBP2+ fibs showed minimal cellular interaction with EC10, both in terms of received and sent signals (Figure 6F,G, Supplementary Figure 3C,D).

### 3.7 Cellular Heterogeneity and Treatment Changes in ST

**FIGURE 7:**
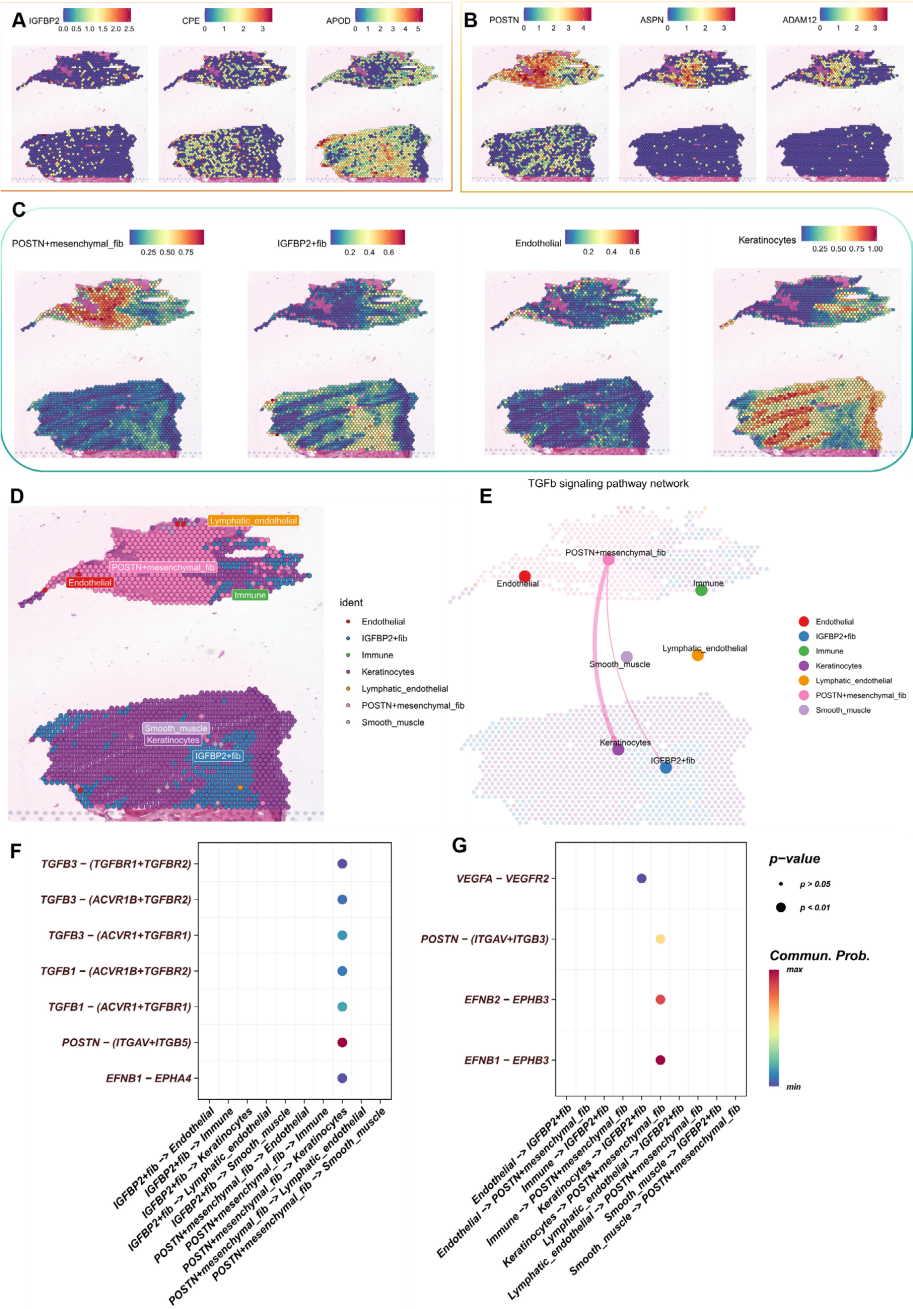
Heterogeneity and cell–cell communication in space. (A, B) Spatial maps of the expression of marker genes for IGFBP2+ fibroblasts (IGFBP2, CPE, and APOD) and POSTN+ mesenchymal fibroblasts (POSTN, ASPN, and ADAM12). (C) Spatial maps of expression derived from different cell types through a deconvolution algorithm. (D) Cellular annotation of each spot in the spatial section based on the deconvolution algorithm. (E) The intensity of spatial TGF‐β signalling interactions between different cells. (F, G) Bubble charts of the two main fibroblast subpopulations (IGFBP2+ fibroblasts and POSTN+ mesenchymal fibroblasts) and their receptor–ligand pairs with other cells in space. Here, the focus is on intercellular signals that have been reported in keloids.

Due to the lack of ST sequencing data for keloids, we collected data from a previous study on AK before and after corticosteroid treatment. Although AK and keloids differ in clinical presentation and underlying mechanisms, they share similarities in the abnormal activation of fibroblasts and abnormal deposition of ECM, which makes our findings also of some reference value for understanding the pathophysiology of keloids. The ST results consist of individual capture locations overlaid on the same tissue section stained with H&E. ST analysis was conducted on two slices of AK specimens before (*N* = 1) and after treatment (*N* = 1). We first analysed the spatial expression of marker genes for IGFBP2+ fibs and POSTN+ mesenchymal fibs. Marker genes for IGFBP2+ fibs (IGFBP2, CPE, and APOD) slightly increased post‐treatment, while those for POSTN+ mesenchymal fibs (POSTN, ASPN, and ADAM12) significantly decreased (Figure 7A,B). Using the RCTD deconvolution method, we estimated cellular heterogeneity and unknown proportions in the spatial transcriptome using scRNA samples containing similar cell types. Results indicated a significant reduction in POSTN+ mesenchymal fibs post‐hormone treatment, while positive cells for IGFBP2+ fibs and keratinocytes relatively increased (Figure 7C). We then defined the cell type represented by each spot based on predicted major cell types (Figure 7D). Finally, CellChat analysis was used to infer potential cellular communication between different cell types in space. The analysis indicated that POSTN+ mesenchymal fibs primarily communicated with keratinocytes via TGF‐β signalling in space (Figure 7E). Compared to IGFBP2+ fibs, POSTN+ mesenchymal fibs exhibited stronger POSTN, TGF‐β, and EPHB ligand–receptor signals with keratinocytes in space (Figure 7F,G).

### 3.8 IGFBP2+ Fibroblasts Contribute to Suppressing Collagen Expression in Keloids

**FIGURE 8:**
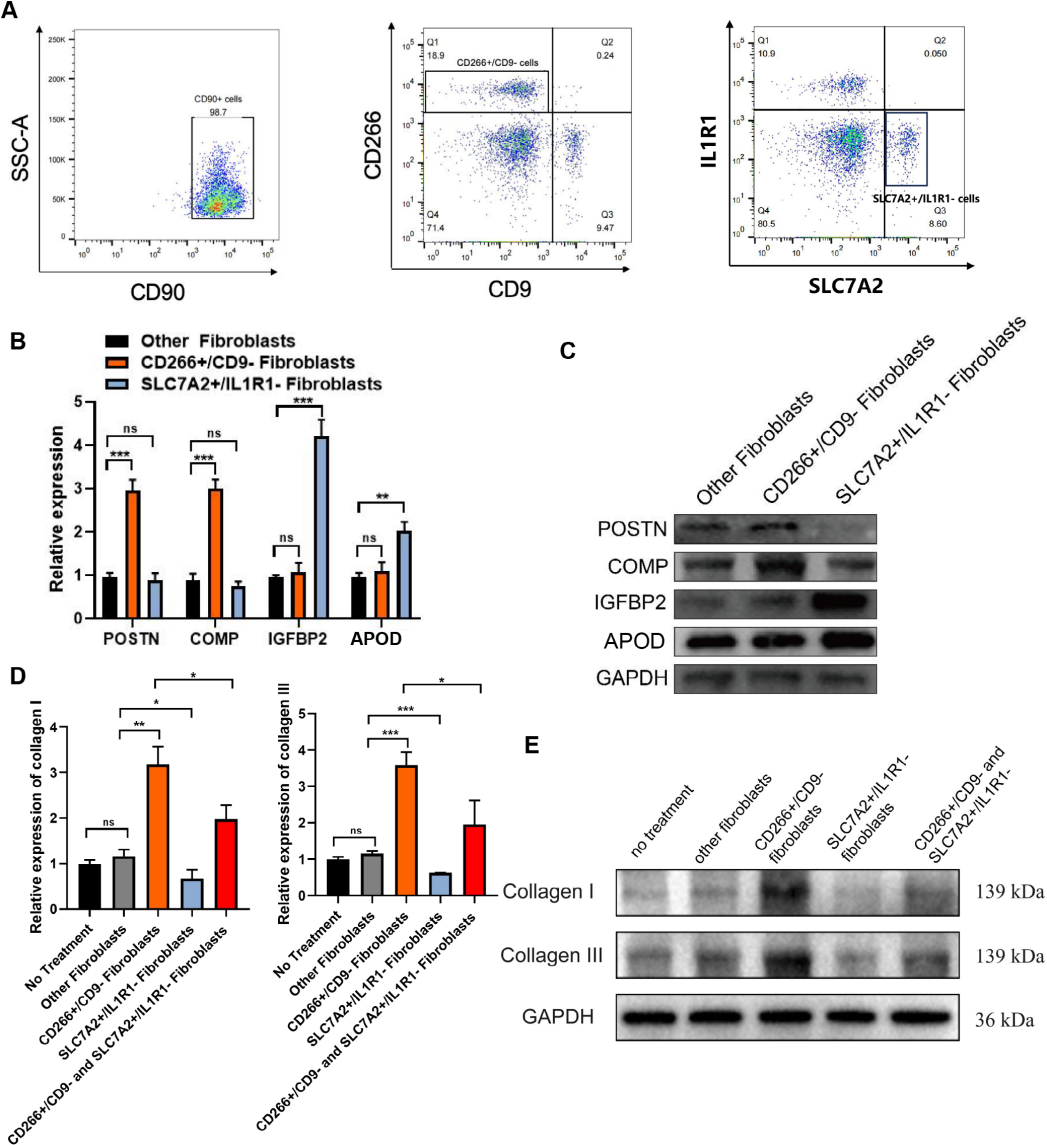
Supernatant from IGFBP2+ fib inhibits fibrosis progression in keloids. (A) Isolation of CD266+/CD9− mesenchymal fibroblasts, SLC7A2+/IL1R1− fibroblasts (IGFBP2+ fibs), and CD90+ other cells (other fibroblasts) from keloid dermis via flow cytometry. (B, C) qRT‐PCR detection of marker gene expression for CD266+/CD9− mesenchymal fibroblasts, SLC7A2+/IL1R1− fibroblasts (IGFBP2+ fibs), and CD90+ other cells (other fibroblasts); Western blot analysis for POSTN, COMP, IGFBP2, and APOD. Data are presented as mean ± SD (*n* = 3 biologically independent experiments). Two‐sided unpaired *t* test, **p < 0.01, ***p < 0.001. Experiments were repeated three times with three different fibroblast donors; representative results are shown here. (D, E) Treatment of ordinary keloid fibroblasts with supernatants from CD266+/CD9− mesenchymal fibroblasts, SLC7A2+/IL1R1− fibroblasts (IGFBP2+ fibs), and CD90+ other cells (other fibroblasts). qRT‐PCR and Western blot analyses were conducted on type I and III collagen expression. Data are presented as mean ± SD (*n* = 3 biologically independent experiments). Two‐sided unpaired *t* test, **p < 0.01, ***p < 0.001. Experiments were repeated three times with three different fibroblast donors; representative results are shown here.

To explore the characteristics and functions of IGFBP2+ fibroblasts in fibrosis, we developed a strategy to isolate these cells. Following the approach of a previous study, we first sorted all CD90+ fibroblasts via flow cytometry and then selected CD266+/CD9− mesenchymal fibroblasts, identified as POSTN+ mesenchymal fibs [20]. In our prior scRNA analysis, we found that most IGFBP2+ fibs highly express SLC7A2 and are negative for IL1R1 expression (Figure 2C and Supplementary Table 3). Consequently, we isolated fibroblasts with IGFBP2+ fib characteristics from non‐mesenchymal fibroblasts using flow sorting. Ultimately, we obtained CD266+/CD9− mesenchymal fibroblasts, SLC7A2+/IL1R1− fibroblasts (IGFBP2+ fibs), and other CD90+ cells (other fibroblasts) (Figure 8A). qRT‐PCR and Western blot confirmed that CD266+/CD9− fibroblasts significantly express marker genes of POSTN+ mesenchymal fibs (POSTN, ASPN, and ADAM12) compared to other fibroblasts. SLC7A2+/IL1R1− fibroblasts significantly expressed IGFBP2+ fib marker genes (IGFBP2, APOD, and FGFBP2) (Figure 8B,C). We then investigated the effects of POSTN+ mesenchymal fibs and IGFBP2+ fibs on collagen expression in keloid fibroblasts. First, we cultured the three cell types (CD266+/CD9−, SLC7A2+/IL1R1−, and other fibroblasts) in dishes. We then collected the supernatants from these fibroblast cultures to treat other normal fibroblasts. qRT‐PCR and Western blot confirmed that collagen I and III expression was highest in the CD266+/CD9− supernatant‐treated group and lowest in the SLC7A2+/IL1R1− supernatant‐treated group. Mixing CD266+/CD9− and SLC7A2+/IL1R1− supernatants before adding them to normal fibroblasts reversed the effects of mesenchymal fibroblasts (Figure 8D,E).

## 4 Discussion

Scar formation is an evolutionary tissue repair mechanism in humans that, while effective to some extent, is difficult to eliminate due to its irreversible nature once formed. After scar tissue replaces the original normal tissue structure, it not only severely affects the functional integrity of the tissue but also significantly impacts aesthetics. Despite significant advances in keloid research in recent years, the core pathological mechanisms remain incompletely understood, and current prevention and treatment strategies for such fibrotic diseases are limited in effectiveness, necessitating the development of innovative therapies. The formation of abnormal fibrotic scars is the result of a pathological wound healing process, and intervening in this process could potentially reduce fibrosis severity and prevent excessive fibrosis [38, 39]. In recent years, researchers have made significant progress in elucidating the fundamental mechanisms by which fibroblasts and myofibroblasts interpret TGF‐β/Smad signalling under pathological conditions, leading to the production of dysregulated collagen and abnormal scar formation [40, 41, 42]. However, multiple fibroblast subpopulations within keloid tissue exhibit significant genetic and phenotypic heterogeneity, and the mechanisms by which this heterogeneity forms and evolves during disease progression remain unclear.

The objective of this study is to apply scRNA‐seq and ST technologies to delve into the cellular heterogeneity and molecular mechanisms of keloids, with a particular focus on the role of fibroblast subpopulations in disease progression, in order to provide a theoretical foundation and scientific evidence for the development of innovative anti‐fibrotic therapeutic strategies. By integrating scRNA‐seq and ST technologies, this study conducts a comprehensive analysis of keloids, focusing specifically on the functions of fibroblast subpopulations in disease progression, with the aim of providing solid scientific evidence and theoretical support for the development of new therapeutic strategies.

In recent years, an increasing body of research evidence has revealed striking similarities between keloids and cancer in multiple aspects, including cellular bioenergetics, genetic and epigenetic changes, and the epithelial‐mesenchymal transition process [43]. Some cancer‐like behaviours exhibited by keloids, such as persistent fibroblast activation and invasive growth into adjacent normal skin, further highlight their similarity to tumour biology [44]. Compared to normal skin, fibroblasts in keloid tissue show higher sensitivity to TGF‐β1, PDGF, and IGF‐I, leading to the overexpression of collagen and other ECM‐related proteins. It is noteworthy that myofibroblasts play a crucial role in the formation and development of keloids; they are not only involved in the synthesis and deposition of collagen but also associated with the activation of various signalling pathways that regulate cell proliferation, differentiation, and apoptosis, thereby affecting the formation of keloids. Due to the key role of myofibroblasts in keloid formation, they are considered an important target for the treatment of keloids. By modulating the activity of myofibroblasts, the formation of keloids can be reduced [45]. These findings not only deepen our understanding of the pathogenesis of keloids but also provide important clues for exploring new therapeutic strategies, especially in the targeted regulation of fibroblast activity and myofibroblast differentiation.

Fibroblasts are central to keloid research, and scRNA‐seq technology offers a data‐driven perspective for a deeper understanding of keloid pathogenesis. This cutting‐edge technology has transformed our traditional understanding of keloid fibroblasts by unveiling their heterogeneity and the existence of multifunctional subtypes during skin inflammatory responses and wound healing processes [46]. Previous studies have categorised dermal fibroblasts into four subgroups: papillary secretory fibroblasts, reticular secretory fibroblasts, mesenchymal fibroblasts, and pro‐inflammatory fibroblasts. Our research builds upon this framework by identifying seven distinct functional fibroblast subpopulations within keloid tissue. Notably, POSTN‐positive mesenchymal fibroblasts (POSTN+ mesenchymal fibs) and IGFBP2‐positive fibroblasts exhibit the most significant differences. Consistent with previous research, we observed an increased proportion of POSTN+ mesenchymal fibs in keloids, closely associated with ECM production [20]. Mesenchymal fibroblasts have been identified as the primary cell type responsible for excessive collagen expression, and their increased numbers may be a critical mechanism in keloid formation. In keloids, the proportion of mesenchymal fibroblasts is significantly higher than in normal scars, and these cells express higher levels of ossification‐related genes such as cartilage oligomeric matrix protein (POSTN) and collagen‐related genes like type I collagen α1 (COL1A1) [47]. Our study further confirms that these cells exhibit high TGF‐β signalling activity and ECM‐related gene expression, indicating their potential central role in the excessive fibrosis process of keloids. Additionally, our research found that POSTN+ mesenchymal fibs exhibit the highest stem cell characteristics, which may be closely related to myofibroblast differentiation—a key step in fibrosis. Mesenchymal stem cells are considered a potential source for myofibroblasts due to their strong self‐renewal capacity, multipotent differentiation potential, and immunomodulatory abilities [48], suggesting that POSTN+ mesenchymal fibs may possess strong self‐renewal and multipotent differentiation potential, contributing to sustained growth in keloids. By analysing ligand–receptor interaction databases, we observed dense communication networks between POSTN+ mesenchymal fibs and other cells, especially within the TGF‐β signalling pathway. Enhanced TGF‐β1/Smad signalling promotes abnormal collagen deposition, increased collagen I/III ratios, and abnormal cross‐linked collagen fibril formation by regulating fibroblast proliferation and apoptosis as well as differentiation into myofibroblasts [38]. In spatial transcriptomic analysis, we further confirmed possible epithelial‐mesenchymal interactions between keratinocytes and mesenchymal fibroblasts. Multiple pieces of evidence suggest that keratinocytes reduce fibroblast apoptosis rates through paracrine signalling [49].

Our research uncovers a unique fibroblast subset, characterised by IGFBP2 positivity (IGFBP2+ fibs), which stands out from conventional mesenchymal subgroups in both phenotype and function. IGFBP2+ fibs are not only less prevalent in keloids but also display reduced collagen expression compared to other fibroblasts. This discovery challenges the prevailing notion that all fibroblasts contribute to fibrosis, indicating that specific subpopulations might possess anti‐fibrotic properties, positioning them as potential therapeutic targets for anti‐fibrotic and anti‐proliferative interventions. IGFBP2+ fibs are distinguished by the expression of marker genes including IGFBP2, FGFBP2, OLFML2A, CPE, APOD, and SLC7A2, which are markedly upregulated in normal skin tissue relative to keloid tissue. Enrichment analysis indicates that IGFBP2+ fibs are predominantly linked to responses to nerve growth factors and the positive regulation of extrinsic apoptotic signalling pathways. These cells also play a role in promoting endothelial cell differentiation, a feature that sets them apart from POSTN+ mesenchymal fibs. In normal skin, IGFBP2+ fibs are engaged in the positive regulation of biomolecule synthesis and cellular metabolism, underscoring their role in preserving skin homeostasis. Interestingly, IGFBP2+ fibs exhibit the least stemness characteristics, suggesting a limited differentiation potential that may be associated with a restricted capacity for tissue repair and regeneration. This could account for their stable presence within normal skin [50]. In terms of cell communication, IGFBP2+ fibs exhibit reduced signal intensity and quantity, particularly showing insensitivity to TGF‐β and POSTN/Periostin signals, a trait that may underlie their anti‐fibrotic effects. Spatial transcriptomic analysis revealed a relative increase in the proportion of IGFBP2+ fibs following hormone treatment, suggesting their involvement in therapeutic responses and reinforcing their potential as therapeutic targets. Finally, our in vitro experiments demonstrated that factors secreted by IGFBP2+ fibs (SLC7A2+/IL1R1−) can suppress collagen expression in other fibroblasts, directly supporting the anti‐fibrotic role of IGFBP2+ fibs. These findings shed new light on keloid treatment strategies, such as augmenting the proportion of IGFBP2+ fibs or replicating their functions to curb excessive fibrosis. However, further investigation is warranted to fully understand the mechanisms underlying IGFBP2+ fibs and their dynamic changes throughout keloid pathogenesis.

This cellular subpopulation is distinguished by a unique gene expression signature, including a range of marker genes such as IGFBP2, FGFBP2, OLFML2A, CPE, APOD, and SLC7A2. The co‐expression of these multiple genes points to a sophisticated regulatory network that likely confers anti‐fibrotic capabilities to this subpopulation in a coordinated manner. Within this network, IGFBP2 appears to be a central player, with other genes potentially contributing through diverse mechanisms to preserve the cell's anti‐fibrotic characteristics. IGFBP2 (IGF binding protein 2) is a crucial element of the IGF system, primarily modulating the activity and bioavailability of IGF‐1 and IGF‐2 by binding to them [51]. IGF binding proteins can target various tissues based on differences in their primary structure and post‐translational modifications, and they can interact with the ECM or cell surfaces via glycoproteins, collagens, and integrins [52]. Notably, previous studies have demonstrated that corneal cells maintain their distinct phenotype when cultured on amniotic stroma and resist differentiation into myofibroblasts, even under TGF‐β stimulation. Amniotic stroma extracts not only sustain the fibroblast phenotype of amniotic stromal cells but can also revert myofibroblasts into fibroblasts [53, 54]. In a recent study, Park et al. hypothesized that IGFBP2 present in keratinocyte‐conditioned media or amniotic extracts might play a significant role in the differentiation and maintenance of corneal fibroblast characteristics. The results indicated that IGFBP2 could inhibit TGF‐β1‐induced upregulation of α‐SMA and enhance the expression of keratin and aldehyde dehydrogenase 1 family member A1 in human corneal fibroblasts [55]. Our study further corroborates, from a bioinformatics standpoint, that IGFBP2 plays a pivotal role in regulating fibroblast differentiation, suggesting that IGFBP2 could be considered a novel anti‐fibrotic strategy in dermatological conditions.

Endothelial‐to‐mesenchymal transition (EndoMT) is a significant factor in keloid formation, with some keloid fibroblasts and myofibroblasts arising from the differentiation of vascular endothelial cells [56]. Our study not only corroborates Shim et al.'s findings on the mesenchymal activation of endothelial cells in keloids but also delves deeper into the complexity and potential mechanisms of this process [19]. Initially, our GSEA revealed that IGFBP2+ fibs and POSTN+ mesenchymal fibs display opposing patterns in regulating endothelial cell differentiation. This finding suggests that different fibroblast subpopulations may have distinct roles in modulating endothelial cell function. IGFBP2+ fibs may foster normal endothelial cell differentiation, while POSTN+ mesenchymal fibs may suppress this process, thereby promoting EndoMT. Our study also uncovered endothelial cell heterogeneity, particularly with the EC10 subpopulation, which exhibits distinct mesenchymal activation characteristics. These cells not only express mesenchymal marker genes but also demonstrate high stemness and differentiation potential similar to POSTN+ mesenchymal fibs. Through CellChat analysis, we identified robust cellular communication between MCTP1+ fibs and EC10, involving signalling pathways such as VEGF, TGF‐β, and EPHB. These signals may act in concert to drive abnormal angiogenesis and the EndoMT process. In contrast, IGFBP2+ fibs have minimal interaction with EC10, consistent with their potential anti‐fibrotic role. These findings offer fresh insights into understanding the mechanisms behind abnormal angiogenesis in keloids.

In this study, we delved into the cellular heterogeneity and molecular mechanisms of keloids, achieving some significant findings, yet some limitations warrant further expansion in our discussion. First, our sample size is limited, and all samples were derived from a single ethnic group, which may restrict the generalizability of our results. Second, the keloid samples we utilised were taken from the central region of the lesions, rather than the peripheral areas, potentially affecting the representativeness of the outcomes. Moreover, with a small sample size (*n* = 6), the generalizability and reliability of our findings are further constrained. Due to the unavailability of spatial transcriptomic data for keloids, we resorted to using data from AK as a substitute. We are aware that AK and keloids differ in their pathophysiology, despite potential clinical similarities. AK is primarily associated with folliculitis, while keloids are related to abnormal wound healing processes post‐skin injury. Therefore, we meticulously differentiated between these two conditions in our analysis and thoroughly discussed the potential biases that may arise from using AK data. Our results indicate that while data from AK can provide some insights into keloids, future studies should directly obtain spatial transcriptomic data for keloids to more accurately explore the anti‐fibrotic mechanisms of IGFBP2+ fibroblasts and leverage this cell subpopulation to develop novel therapeutic strategies. Additionally, further investigation into the activation mechanisms of POSTN+ mesenchymal fibroblasts, as well as the interaction mechanisms between fibroblast subpopulations and other cell types, especially at various stages of keloid development, is necessary.

## 5 Conclusion

This study has uncovered the cellular diversity and molecular mechanisms of keloids through single‐cell and ST analysis, identifying the key roles of POSTN‐positive and IGFBP2‐positive fibroblast subsets. The POSTN‐positive subset is associated with excessive ECM production in keloids, while the IGFBP2‐positive subset, more prevalent in normal skin, exhibits anti‐fibrotic characteristics. The research also highlights the significance of endothelial cell heterogeneity and its interaction with fibroblasts, suggesting a role for EndoMT in keloid formation. These findings lay the groundwork for developing new therapeutic strategies, particularly targeting IGFBP2‐positive fibroblasts as potential therapeutic targets.

## Author Contributions

Research design: Songyun Zhao, Jiaheng Xie, Qian Zhang, and Tianyi Ni. Data collection: Jinde Lin, Weicheng Gao, Zhaoli Ping, Min Yi, Liying Tu, Pengpeng Zhang, Dan Wu, Qikai Tang, Chenfeng Ma, and Yucang He. Data analysis: Jiaheng Xie and Tianyi Ni. Manuscript preparation: Songyun Zhao, Liqun Li, Guoping Wu, and Wei Yan. Manuscript editing: Songyun Zhao, Liqun Li, Guoping Wu, and Wei Yan. All authors contributed to the article and approved the submitted version.

## Ethics Statement

This study was approved by the Medical Ethics Committee of the First Affiliated Hospital of Nanjing Medical University and the Affiliated Friendship Plastic Surgery Hospital (Approval No: 2024‐SR‐383). Informed consent was obtained from each patient before participation, and all participants were of Han ethnicity. The study was conducted under local legal and institutional requirements.

## Conflicts of Interest

The authors declare no conflicts of interest.

## Supporting information


**SUPPLEMENTARY FIGURE 1** Heterogeneity of cells in keloids. (A–C) UMAP plots of single‐cell features coloured by histological type, sample and cluster of 24 cells in this study. (D) Identification of unique marker genes representing each cell subpopulation. (E) UMAP plot of the final 10 cell types identified. (F) Genes up‐ and down‐regulated in each cell type in scars compared to normal skin.

**SUPPLEMENTARY FIGURE 2** Heterogeneity of fibroblasts in keloids. (A–C) UMAP plots of fibroblast characteristics by histological type, sample and 7 cell clusters coloured in this study. (D) Comparison of representative differentially expressed genes between keloid mesenchymal fibroblasts and normal skin mesenchymal fibroblasts.

**SUPPLEMENTARY FIGURE 3** Cellular communication between endothelial cells and fibroblasts. (A, B) Circle plots of the strength and number of interactions between seven fibroblast subpopulations and EC10. (C, D) Intensity of all ligand–receptor pairs between EC10 and two fibroblast subpopulations.

**SUPPLEMENTARY TABLE 1** clinical information on the patients.

**SUPPLEMENTARY TABLE 2** Primer sequences used in this study.

**SUPPLEMENTARY TABLE 3** Differentially expressed genes in all fibroblast subtypes.

## Data Availability

The data that support the findings of this study are available from the corresponding author upon reasonable request.
