# Supplementary figures and images for: New Anti‐Fibrotic Strategies for Keloids: Insights From Single‐Cell Multi‐Omics
Source: Cell Prolif. 2025 Feb 4;58(6):e13818. doi: 10.1111/cpr.13818 (PMC12179555; doi:10.1111/cpr.13818)

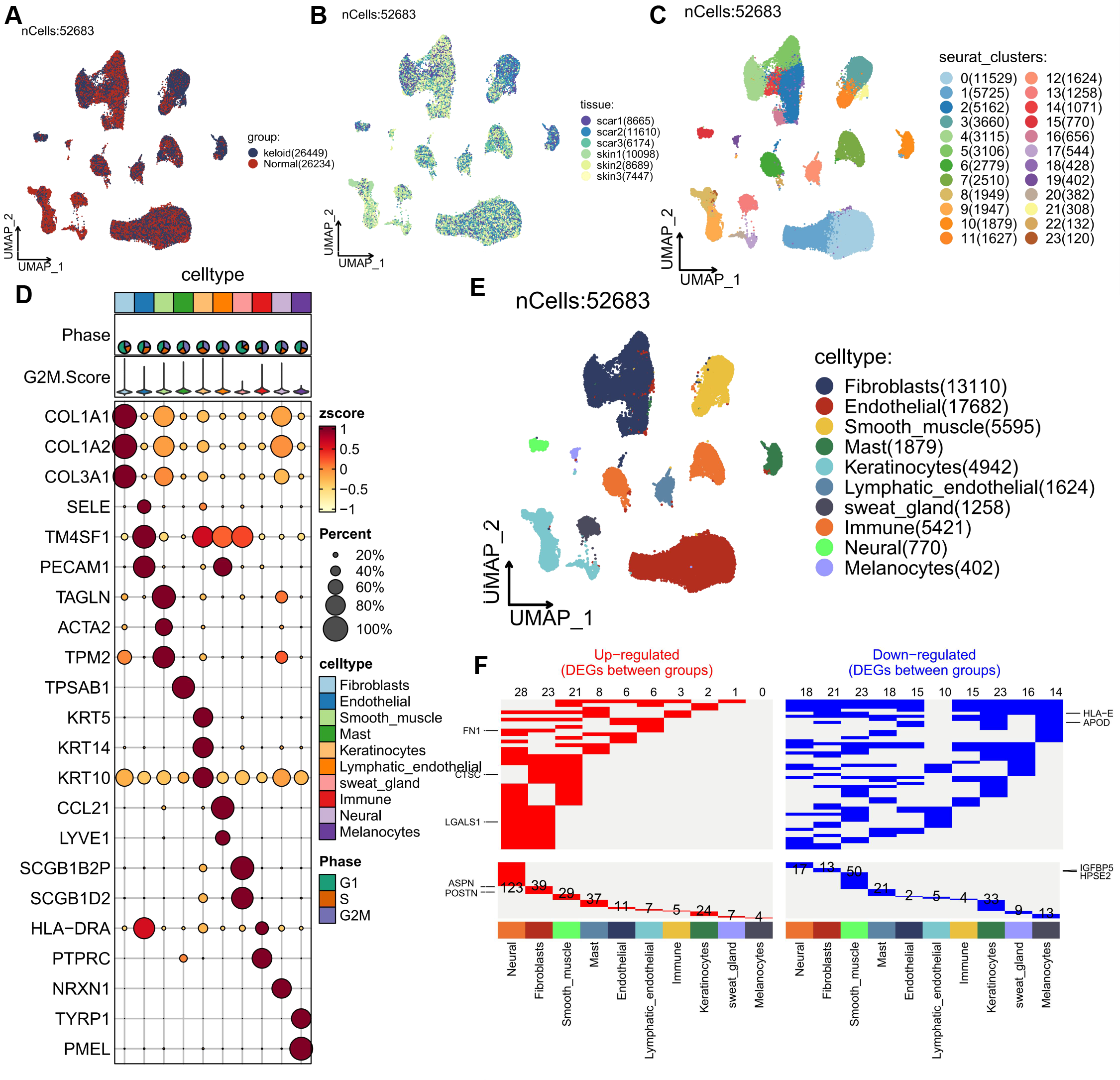

Supplement: Supplementary file 1 — SUPPLEMENTARY FIGURE 1 Heterogeneity of cells in keloids. (A–C) UMAP plots of single‐cell features coloured by histological type, sample and cluster of 24 cells in this study. (D) Identification of unique marker genes representing each cell subpopulation. (E) UMAP plot of the final 10 cell types identified. (F) Genes up‐ and down‐regulated in each cell type in scars compared to normal skin. [file CPR-58-e13818-s002.tif]

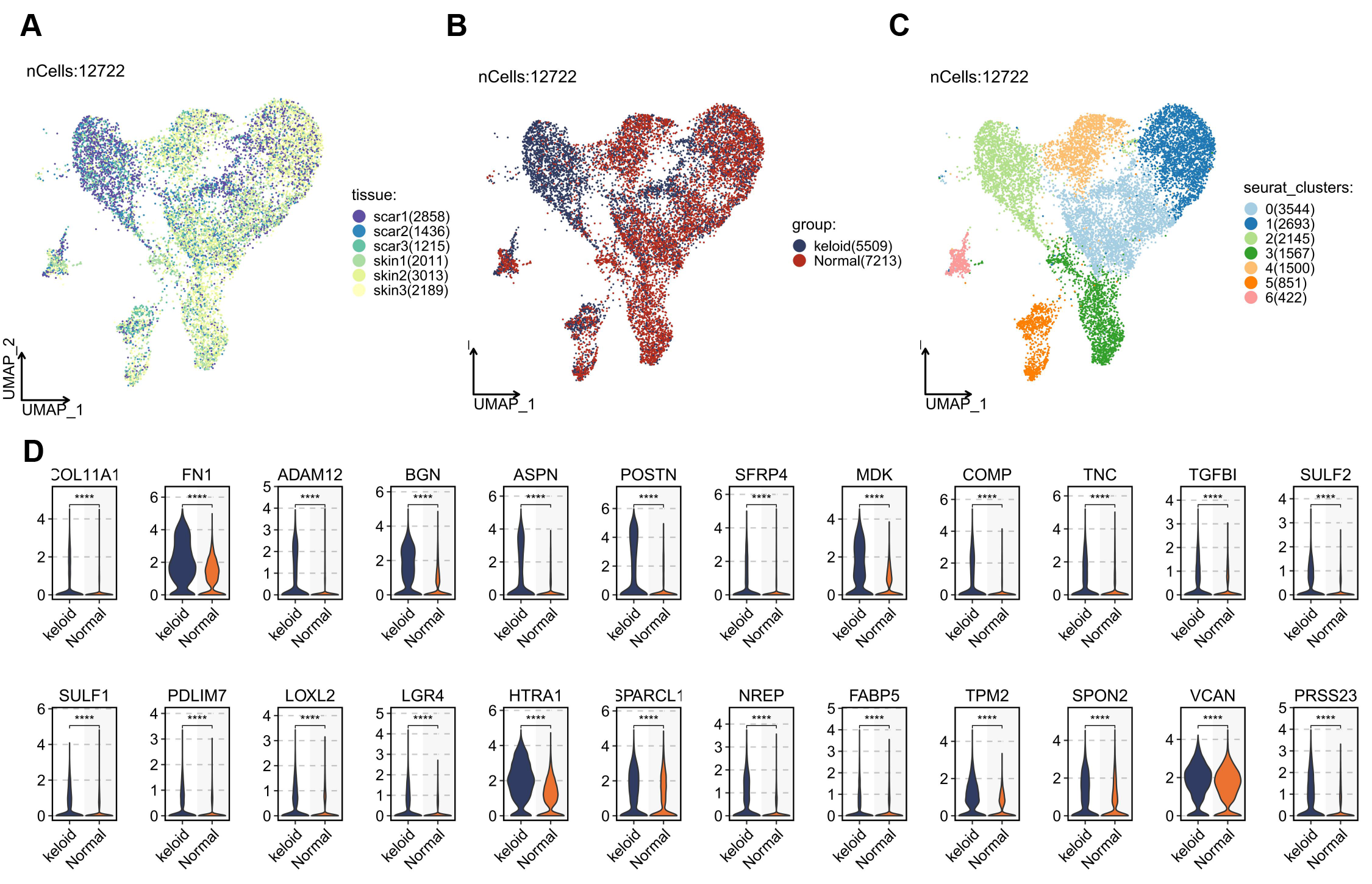

Supplement: Supplementary file 2 — SUPPLEMENTARY FIGURE 2 Heterogeneity of fibroblasts in keloids. (A–C) UMAP plots of fibroblast characteristics by histological type, sample and 7 cell clusters coloured in this study. (D) Comparison of representative differentially expressed genes between keloid mesenchymal fibroblasts and normal skin mesenchymal fibroblasts. [file CPR-58-e13818-s003.tif]

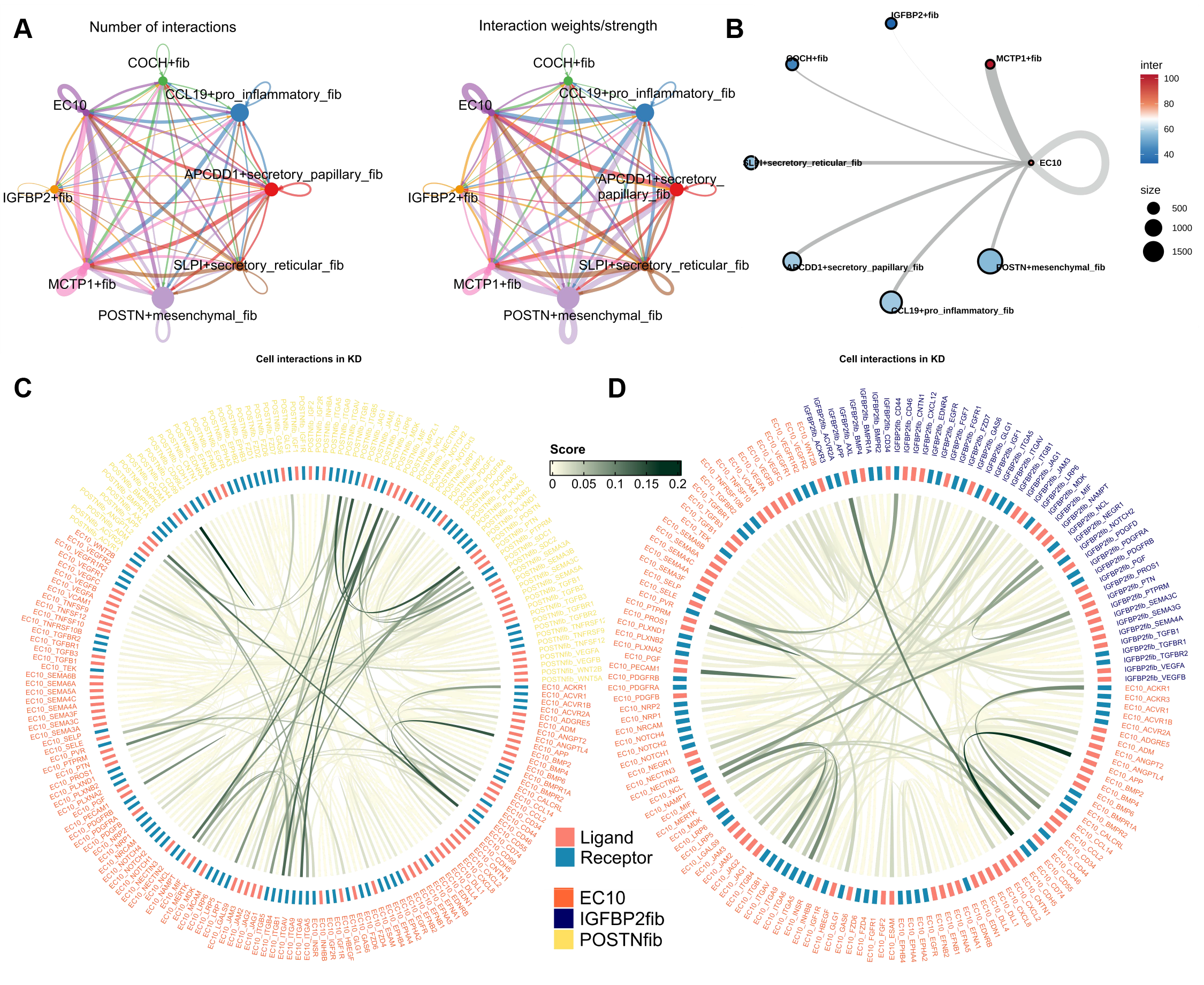

Supplement: Supplementary file 3 — SUPPLEMENTARY FIGURE 3 Cellular communication between endothelial cells and fibroblasts. (A, B) Circle plots of the strength and number of interactions between seven fibroblast subpopulations and EC10. (C, D) Intensity of all ligand–receptor pairs between EC10 and two fibroblast subpopulations. [file CPR-58-e13818-s001.tif]
